# Profiles of cognitive impairment in chronic heart failure—A cluster analytic approach

**DOI:** 10.3389/fnhum.2023.1126553

**Published:** 2023-04-20

**Authors:** Dennis Göpfert, Jan Traub, Roxane Sell, György A. Homola, Marius Vogt, Mirko Pham, Stefan Frantz, Stefan Störk, Guido Stoll, Anna Frey

**Affiliations:** ^1^Comprehensive Heart Failure Center, University Hospital Würzburg, Würzburg, Germany; ^2^Department of Medicine I, University Hospital Würzburg, Würzburg, Germany; ^3^Department of Psychiatry, Psychosomatics, Psychotherapy, University Hospital Würzburg, Würzburg, Germany; ^4^Department of Neuroradiology, University Hospital Würzburg, Würzburg, Germany; ^5^Department of Neurology, University Hospital Würzburg, Würzburg, Germany

**Keywords:** chronic heart failure, cluster analysis, cognitive impairment, intensity of attention, glial fibrillary acidic protein

## Abstract

**Background:**

Cognitive impairment is a major comorbidity in patients with chronic heart failure (HF) with a wide range of phenotypes. In this study, we aimed to identify and compare different clusters of cognitive deficits.

**Methods:**

The prospective cohort study “Cognition.Matters-HF” recruited 147 chronic HF patients (aged 64.5 ± 10.8 years; 16.2% female) of any etiology. All patients underwent extensive neuropsychological testing. We performed a hierarchical cluster analysis of the cognitive domains, such as intensity of attention, visual/verbal memory, and executive function. Generated clusters were compared exploratively with respect to the results of cardiological, neurological, and neuroradiological examinations without correction for multiple testing.

**Results:**

Dendrogram and the scree plot suggested three distinct cognitive profiles: In the first cluster, 42 patients (28.6%) performed without any deficits in all domains. Exclusively, the intensity of attention deficits was seen in the second cluster, including 55 patients (37.4%). A third cluster with 50 patients (34.0%) was characterized by deficits in all cognitive domains. Age (*p* = 0.163) and typical clinical markers of chronic HF, such as ejection fraction (*p* = 0.222), 6-min walking test distance (*p* = 0.138), NT-proBNP (*p* = 0.364), and New York Heart Association class (*p* = 0.868) did not differ between clusters. However, we observed that women (*p* = 0.012) and patients with previous cardiac valve surgery (*p* = 0.005) prevailed in the “global deficits” cluster and the “no deficits” group had a lower prevalence of underlying arterial hypertension (*p* = 0.029). Total brain volume (*p* = 0.017) was smaller in the global deficit cluster, and serum levels of glial fibrillary acidic protein were increased (*p* = 0.048).

**Conclusion:**

Apart from cognitively healthy and globally impaired HF patients, we identified a group with deficits only in the intensity of attention. Women and patients with previous cardiac valve surgery are at risk for global cognitive impairment when suffering HF and could benefit from special multimodal treatment addressing the psychosocial condition.

## 1. Introduction

Due to the aging population and improved survival after myocardial infarction, the prevalence of chronic heart failure (HF) is constantly increasing (Ponikowski et al., 2016). Envisaged holistic care includes the identification and targeted treatment of secondary comorbidities (Ponikowski et al., 2016). Multiple cross-sectional studies described relevant cognitive impairment (CI) in almost every second HF patient, which relates to the adverse outcomes and increased healthcare costs (Sauve et al., 2009; Almeida et al., 2012; Frey et al., 2018). While the etiology of CI in HF is complex and subject to current research, CI mostly affects the domains of the intensity of attention, memory, and executive functions (Wolfe et al., 2006; Vogels et al., 2007; Okonkwo et al., 2010; Pressler et al., 2011). It has been shown that the extent of these deficits correlates with the severity of chronic HF and varies in the degree of stability, ranging from reversible CI to chronic courses (Dardiotis et al., 2012). Because of these complexities, neuropsychological assessment of subtle, subclinical cognitive changes is of great importance. From a therapeutic perspective, early intervention could help to halt CI deterioration and the development of dementia. However, little is known about the individual cognitive profiles and their clinical or prognostic importance in HF patients (Pullicino and Hart, 2001).

Therefore, this *post hoc* analysis aims to characterize distinct patterns of CI, identify their clinical characteristics, and evaluate their prognostic impact. We performed hierarchical clustering of chronic HF patients according to their performance in neuropsychological testing and analyzed clinical, laboratory, and apparative data of 147 patients within the comprehensive Cognition.Matters-HF study.

## 2. Materials and methods

### 2.1. Study design and examinations

The interdisciplinary, investigator-initiated, single-centered, prospective cohort study “Cognition.Matters-HF” was approved by the local Ethics Committee (study registration number 245/10) and complies with the Declaration of Helsinki, as previously published (Frey et al., 2018). Patients with stable chronic systolic or diastolic HF without major psychiatric or neurologic disorders were eligible (Supplementary Table 1). All patients underwent an extensive interdisciplinary workup according to a prespecified protocol. Examinations included cardiovascular and neurological clinical examination, neuropsychological test battery, electrocardiogram, echocardiography, 6-min walk testing, and brain magnetic resonance imaging (MRI). We measured routine blood parameters and the neurodegenerative serum biomarkers neurofilament light chain (NfL), glial fibrillary acidic protein (GFAP), and phosphorylated Tau protein (pTau) according to a prespecified protocol from venous blood (Traub et al., 2022).

### 2.2. Neuropsychological testing

Patients underwent an extensive neuropsychological test battery (Supplementary Table 2) with a duration of 1 h between 9 and 11 a.m. to guard against circadian power fluctuation. The test outputs are given as standardized *t*-values to account for the modifying effect of age, sex, and educational level. Test Battery of Attentional Performance quantified intensity and selectivity of attention. The intensity of attention refers to the focus on and maintenance of the given tasks. For visual/verbal memory and working memory, Visual and Verbal Memory Test, Digit Span Forward, and Block Tapping Span Forward tests were applied. Visual/verbal fluency was analyzed using Regensburger Word Fluency Test and HAMASCH-5-Point-Test (Table 1). The reliability of tests ranged between 0.60 and 0.99 (Schellig and Schächtele, 2009). Executive function comprised the domains selectivity of attention, working memory, and visual/verbal fluency. The test battery was compiled by a trained neuropsychologist in an elaborate process. To reduce any form of bias, the tasks were not only spoken to the patients but also played to them in a standardized way.

**Table 1:** Distribution of neuropsychological tests and assessed variables according to subdivision into the three cognitive areas intensity of attention, memory, and executive functions.

**Test**	**Cognitive domain/specification**	***T*-value**
	**Intensity of attention**	
TAP Alertness	Alertness, reaction times	Median of reaction times with and without previous acoustic signal
	**Memory**	
VVM 2 map	Short- and medium-term visual memory	Number of correct crosses
VVM 2 text	Short- and medium-term verbal memory	Number of correct responses
	**Executive functions**	
TAP GoNoGo	Selectivity of attention (focusing, response inhibition)	Number of errors
TAP Divided Attention	Selectivity of attention (dividing)	Number of errors
WMS-R digit span	Verbal working memory	Number of correctly remembered series
WMS-R block tapping span	Visual working memory	Number of correctly touched series
RWT lexically change of categories	Verbal fluency (spontaneous cognitive flexibility, shifting)	Number of correct words
H5PT	Visual fluency (spontaneous cognitive flexibility)	Number of correct patterns

### 2.3. Generation of clusters

**Figure 1:**
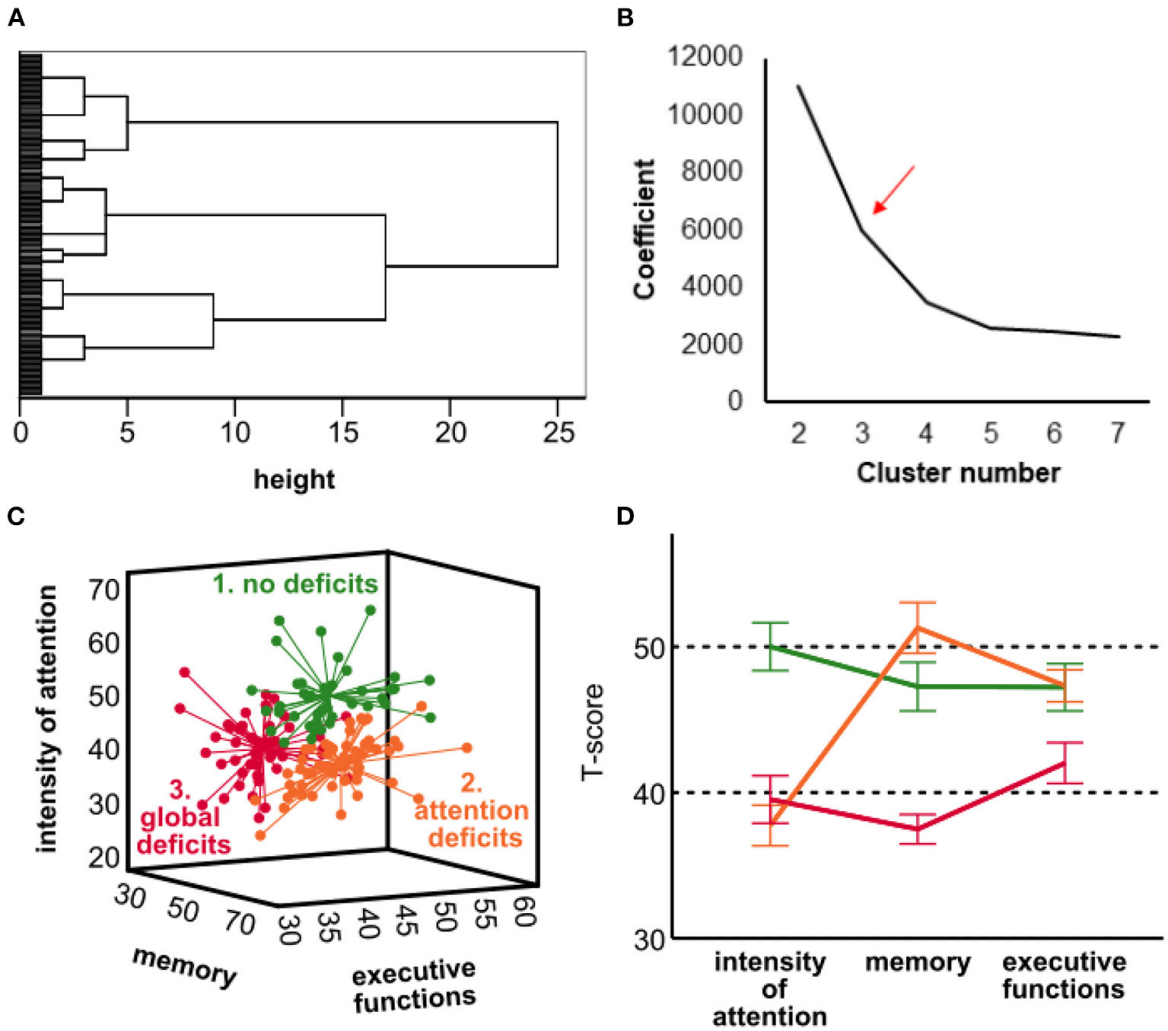
Visualization of cognitive clustering. **(A)** Dendrogram for hierarchical representation of clusters. Each leaf represents an individual observation. Leaves are spaced evenly along the vertical axis. The horizontal axis indicates a distance or dissimilarity measure. The height of a node represents the distance between the two clusters. The graph is used to visualize how clusters are formed. The number of clusters that seemed to have an optimal dissimilarity measure within each cluster was set at three. **(B)** Scree plot derived from hierarchical cluster analysis. The *y*-axis represents the degree of homogeneity within the cluster, while the *x*-axis shows the number of clusters. The optimal number was set at the bend of the curve, which also was at three clusters confirming visual determination by our dendrogram. **(C)** Three-dimensional visualization and line diagram of cognitive clusters as generated by subsequent k-means cluster analysis. Each point represents an HF patient. Lines represent the central trend of the cases in terms of their cognition. **(D)** T-scores of the cognitive domains are given. Dots represent a single patient; centroid projection was applied. Whiskers display mean and 95% confidence intervals.

We performed hierarchical clustering according to cognitive function using Ward's method and squared Euclidean distance as a similarity measure of the three variables intensity of attention, memory, and executive functions. For each cluster number, the sum of squares was calculated by the statistical program. The curve of the sum of squares was plotted according to the number of clusters (Figure 1B). The location of a bend (knee) in the plot is generally considered an indicator of the appropriate number of clusters (Hunt and Jorgensen, 2011; Prasanna and Vijaya, 2022).

### 2.4. Cerebral MRI and processing

Brain MRI was performed on a 3 Tesla scanner (Siemens MAGNETOM Trio, Erlangen, Germany) using a 12-channel head coil. Sequences included T1W FLASH, T1W 3D TFL, T2W FLAIR, T2W TSE, DWI, localizers, SV spectroscopy, and ASL perfusion (Supplementary Table 3). Cerebral atrophy was rated visually on a scale from 1 to 8, medial temporal lobe atrophy by Scheltens's score from 0 (normal) to 4 (severe atrophy), and white matter hyperintensities (WMH) using Fazekas score, ranging from 0 to 3. Mean Scheltens's score ≥1.5 in patients younger than 75 years and ≥2 in patients older than 75 years was considered pathological (Frey et al., 2018). MRI analysis was performed according to the study protocols of ASPS and ASPS-Fam. The MRI recordings were made semi-quantitatively. Images were read and documented by an expert neuroradiological doctor. The findings were formally approved by a second senior neuroradiologist. Afterward, the available imaging data were compared with the available imaging data of healthy controls. Cerebral scores were rated visually. The investigator had no access to further data of the patients. There was no further calculation for inter-rater reliability (Frey et al., 2018).

### 2.5. Statistical analysis

Variables are depicted as median (interquartile range) for metric and interval scaled data or count (percentage) for ordinal and nominal data. ANOVA test or *t*-test was used. In the absence of a normal distribution or violation of other assumptions, non-parametric procedures such as the Kruskal–Wallis or the Mann–Whitney *U*-test were used. If Levene's test was significant, indicating missing variance homogeneity between groups, Welch's *t*-test was used. *Post hoc* analysis included Scheffé's test for variables with homogeneous variance and Dunnett's T3 test for those without. For nominally scaled variables, the chi-squared test or Fisher's exact test was used, the latter was taken into account when sample sizes within cells were below five. The statistical software package SPSS, version 27, was used.

## 3. Results

### 3.1. Clinical characteristics of the study cohort

The comprehensive baseline characteristics of 148 included chronic HF patients within the Cognition.Matters-HF study have been previously described in detail (Frey et al., 2018) and are provided in Table 2 and Supplementary Table 4. The complete neuropsychological examination was available in 147 participants, aged 63.9 ± 10.8 years; 23 patients were female (15.6%). The severity of HF symptoms was mild in the majority of patients, with 77% in NYHA functional class I or II. The most common reason for HF was coronary artery disease (65%). The mean 6-min walking distance was 392 ± 99 m, while the mean left ventricular ejection fraction was 42.5 ± 8.1%. The prevalence of deficits in at least one cognitive domain was 59.9%.

**Table 2:** Patient characteristics according to cognitive clusters.

**Parameter**	**All patients (*n* = 147)**	**No deficits (1) (*n* = 42)**	**Attention deficits (2) (*n* = 55)**	**Global deficits (3) (*n* = 50)**	***P*-value**
**Cognitive domains**					
Intensity of attention	41.5 (36.5–47.0)	49.3 (46.4–52.5)	38.5 (34.5–41.5)	39.5 (35.9–44.0)	**0.001** ^**1>2, 1>3**^
Memory (visual/verbal)	45.3 (39.5–51.5)	46.1 (43.7–51.1)	51.5 (46.0–54.3)	38.8 (34.3–39.8)	**0.001** ^**1>3, 2>3**^
Executive functions	44.8 (41.7–49.7)	46.7 (42.8–52.2)	46.8 (44.5–50.3)	41.1 (38.8–44.1)	**0.001** ^**1>3, 2>3**^
**Sociodemographic**					
Age (years)	65 (56–72)	66 (54–75)	63 (54–70)	68 (58–74)	0.163
Female sex	23 (15)	4 (10)	5 (9)	14 (28)	**0.012** ^**3>2**^
Body mass index (kg/m^2^)	28 (26–32)	28 (25–31)	29 (26–33)	28 (26–31)	0.603
Education level (university entrance qualification)	29 (20)	8 (19)	13 (24)	8 (16)	0.491
**Cardiovascular**					
Heart rate (bpm)	63 (58–70)	60 (57–71)	62 (57–67)	68 (59–75)	**0.035** ^**3>2**^
Systolic blood pressure (mmHg)	136 (125–151)	135 (120–151)	138 (125–153)	138 (126–149)	0.775
Diastolic blood pressure (mmHg)	80 (75–89)	80 (75–85)	84 (73–90)	80 (75–89)	0.677
NYHA class I	41 (28)	13 (31)	13 (24)	15 (30)	0.868
NYHA class II	87 (59)	25 (60)	34 (62)	28 (56)	0.868
NYHA class III	19 (13)	4 (10)	8 (15)	7 (14)	0.868
Length of HF diagnosis (years)	4.0 (1.0–10.0)	6.0 (1.8–11.3)	4.0 (1.0–9.0)	4.0 (1.0–10.3)	0.487
6-min walking test distance (m)	400 (340–460)	420 (360–480)	420 (350–460)	380 (305–440)	0.138
Left ventricular ejection fraction (%)	44 (38–48)	42 (35–47)	45 (41–48)	44 (36–48)	0.222
NT-proBNP (pg/ml)	672 (237–1,677)	680 (254–1,907)	435 (154–1,036)	879 (412–1,853)	0.364
Hemoglobin A1c (%)	6.0 (5.5–6.6)	6.0 (5.5–6.5)	6.0 (5.5–6.6)	5.9 (5.5–6.6)	0.133
Low-density lipoprotein (md/dl)	98.5 (78.0–123.3)	100.5 (77.8–123.5)	97.5 (74.3–119.8)	100.0 (82.0–134.0)	0.168
Ischemic heart failure	95 (64)	26 (61)	39 (70)	30 (60)	0.460
Guideline-based heart failure therapy	124 (84)	36 (86)	48 (87)	40 (80)	0.568
**Risk factors**					
Diabetes mellitus type II	43 (29)	11 (26)	19 (35)	13 (26)	0.551
Arterial hypertension	118 (80)	28 (67)	48 (87)	42 (84)	**0.029** ^**2>1**^
Hyperlipidemia	106 (72)	33 (79)	43 (78)	30 (60)	0.063
(Former) smoking	88 (60)	31 (74)	33 (60)	24 (48)	**0.042** ^**1>3**^
**Comorbidities**					
Coronary artery disease	99 (67)	29 (69)	39 (71)	31 (62)	0.600
History of myocardial infarction	79 (54)	24 (57)	34 (62)	21 (42)	0.110
Atrial fibrillation	34 (23)	14 (33)	7 (13)	13 (26)	**0.049** ^**1>2**^
**Previous interventions**					
Coronary revascularization	70 (48)	21 (50)	30 (55)	19 (38)	0.288
Valvular operations	10 (7)	0 (0)	2 (4)	8 (16)	**0.005** ^**3>1**^
Reanimation/defibrillation	8 (5)	1 (2)	5 (9)	2 (4)	0.386

Count (percentage) and median (interquartile range) are shown. Significant differences in *post hoc* analyses are indicated by greater than (>) and less than (<) sign. HF, heart failure; NYHA, New York Heart Association. Bold numbers indicate values equal to or below a significance level of *p* = 0.05.

### 3.2. Generation and description of cognitive clusters

The cluster analysis is based on composite T-scores of the three cognitive domains intensity of attention, memory, and executive functions. We performed hierarchical agglomerative cluster analysis using Ward's method and the squared Euclidean distance. Examining the dendrogram and the scree plot (Figure 1) suggested a three-cluster solution. Ward's method clustering rendered a first group, which comprised 42 patients (28.6%), showed nearly normal performance in all three domains of cognition and was therefore labeled “no deficits” (ND). A total of 55 HF patients (37.4%) in a second “attention deficit” (AD) cluster showed only selective deficits in the domain intensity of attention. The third cluster included 50 patients (34.0%) and was characterized by deficits in all domains (“global deficits”; GD). As a proof of concept, we found significant differences in all three cognitive domains between all clusters (Supplementary Table 4). To investigate the particular impairment of intensity of attention in the second cluster, we also analyzed how the cognitive domains related to each other (Supplementary Figure 1). While the T-scores of visual/verbal memory correlated significantly positively with executive functions, the intensity of attention did not correlate to any of the other two domains. We also compared the total cohort to the norm of a T-score of 50 for all cognitive domains and found a significant difference of *p* < 0.001.

### 3.3. Clinical comparison of clusters

Next, we aimed to detect clinical differences between generated cognitive clusters in an exploratory approach. As depicted in Table 2, chronic HF parameters such as 6-min walking distance, New York Heart Association Class, NT-proBNP, and left ventricular ejection fraction did not differ between clusters. While age also did not diverge between clusters, the proportion of women in the clusters differed significantly with the highest percentages in the GD cluster. This group also had a higher proportion of valve surgeries and tended to have lower hemoglobin levels (*p* = 0.035). Similarly, lower mean corpuscular hemoglobin concentration (*p* = 0.043) and higher heart rate (*p* = 0.035) could be differentiated. Atrial fibrillation was lowest in the “attention deficits” cluster. Exclusively for the cognitively intact cluster, a lower proportion of patients with arterial hypertension could be determined. There was no difference in education level (university entrance qualification) between all clusters considered (*p* = 0.491).

Further detailed analysis (Supplementary Table 3) revealed no relevant differences between clusters in other echocardiographic and laboratory parameters.

### 3.4. Brain imaging data and neurological biomarkers

In cerebral MRI, visually rated cerebral and periventricular, and white matter hyperintensities did not differ between groups (Table 3). Equally, the frequency of cerebral ischemia did not differ with respect to territorial, border zones, or lacunar infarcts of the medullary camp. Further evaluation revealed reduced total brain volume (*p* = 0.017) in the globally deficient cluster.

**Table 3:** Neurochemical and neuroradiological measures according to cognitive clusters.

**Parameter**	**All patients (*n* = 147)**	**No deficits (*n* = 42)**	**Attention deficits (*n* = 55)**	**Global deficits (*n* = 50)**	***P*-value**
**Neuronal biomarkers**					
Glial fibrillary acidic protein (pg/ml)	247 (164–380)	226 (137–396)	212 (155–348)	307 (194–398)	**0.048** ^**3>2**^
Neurofilament light chain (pg/ml)	26.2 (17.0–41.6)	22.5 (14.5–35.3)	27.5 (15.2–42.7)	28.5 (22.6–40.7)	0.127
Phosphorylated tau protein (pg/ml)	1.63 (1.08–2.50)	1.69 (1.04–2.36)	1.38 (0.98–2.40)	1.74 (1.22–3.01)	0.183
**MRI parameters**					
Lacunar infarction (%)	9 (6.7)	4 (9.7)	2 (3.8)	3 (6.3)	0.331
Territorial infarction (%)	2 (1.4)	2 (5.1)	0 (0.0)	0 (0.0)	0.081
Microinfarctions global (%)	(38)	17 (42)	22 (41)	16 (33)	0.810
Total brain volume (ml)	1,184 (1,116–1,251)	1,191 (1,135–1,264)	1,198 (1,137–1,269)	1,136 (1,100–1,225)	**0.017** ^**1>3**^
White matter hyperintensity score (0–3)	1 (1–1)	1.00 (1.00)	1.00 (1.00)	1.00 (1.00)	0.232
White matter hyperintensity volume (mm3)	2.66 (1.55–4.70)	2.79 (1.40–5.07)	2.48 (1.52–3.84)	2.88 (4.99)	0.318
Periventricular hyperintensity score (0–3)	1.00 (1.00–1.00)	1.00 (1.00)	1.00 (1.00)	1.00 (1.75)	0.818
Outer cerebral atrophy score (0–8)	3.00 (2.00–3.25)	3.00 (2.00–4.00)	3.00 (2.00–3.00)	3.00 (2.00–3.00)	0.778
Inner cerebral atrophy score (0–8)	3.00 (2.00–4.00)	3.00 (2.00–5.00)	3.00 (2.00–4.00)	3.00 (3.00–4.00)	0.835
Cerebral atrophy score total (0–8)	3.00 (2.50–4.00)	3.00 (2.00–4.75)	3.00 (2.25–4.00)	3.00 (2.50–3.50)	0.820
Hippocampal atrophy right (0–4)	2.00 (1.00–3.00)	2.00 (1.00–2.00)	2.00 (1.00–2.00)	2.00 (1.00–3.00)	0.233
Hippocampal atrophy left (0–4)	2.00 (1.00–3.00)	2.00 (1.00–3.00)	2.00 (1.00–3.00)	2.50 (2.00–3.00)	0.221
Hippocampal atrophy total (0–4)	2.00 (1.38–2.63)	2.00 (1.00–2.50)	2.00 (1.00–2.50)	2.00 (1.50–3.00)	0.179
Pathological Scheltens' score	106 (74.6)	28 (68.3)	37 (69.8)	41 (85.4)	0.107

Count (percentage) and median (interquartile range) are shown. Significant differences in *post hoc* analyses are indicated by greater than (>) and less than (<) sign. Bold numbers indicate values equal to or below a significance level of *p* = 0.05.

In the analysis of serum biomarkers for central nervous system damage (Table 3), serum GFAP concentrations were elevated in the globally deficient cluster (347 ± 231 pg/ml) compared with the other clusters (*p* = 0.048). NfL and pTau did not differ within the groups.

## 4. Discussion

The current analysis is based on 147 chronic HF patients in the Cognition.Matters-HF study. The incidence of CI in HF patients in at least one domain was 59.9% in this cohort, which is broadly in line with several other publications (Dardiotis et al., 2012). Several key findings emerged: We identified three distinct cognitive clusters with none, global, and only intensity of attention deficits. Clinical differences were marginal between clusters, with differences in women share, previous heart valve surgery, and hypertension. Global cognitive deficit associated with higher serum GFAP and global brain atrophy.

### 4.1. Cognitive clusters in chronic HF

As a principal finding, we propose a division into cognitively intact, the intensity of attention, and globally deficient HF patients in a three-cluster solution. These findings partly contrast a publication from 2012, which suggested the three clusters “cognitively normal,” “memory deficient,” and “globally deficient” (Miller et al., 2012). While patients with intact cognition and only intensity of attention deficits showed hardly any clinical differences, patients with global deficits were more likely to be female, have had more heart valve surgery, and tended to have lower brain volumes. Elevated serum GFAP in this cluster parallels these findings.

### 4.2. Intensity of attention deficits

Attention deficit itself is a known consequence of chronic HF (Alosco et al., 2012b). The here-described selective intensity of attention deficits in a relevant proportion of patients suggests that this area behaves independently of others. The cross-sectional approach of this analysis limits further interpretation. It would be interesting to investigate whether the intensity of attention deficits may be early signs of global CI or represent a completely independent signature of CI. However, it has been shown that clinical treatment can reverse the intensity of attention deficits in chronic HF (Almeida and Tamai, 2001), suggesting unstable, fluid performance in this domain. Based on this, it is debatable whether attention is such an unstable measure that it may not be ideally suited to adequately assess global cognition deficits in HF patients over the long term.

### 4.3. Clinical characteristics of generated clusters

Surprisingly, none of the parameters clinically associated with HF differed between clusters. This is in marked contrast to many other publications where left ventricular ejection fraction, NT-proBNP, NYHA class, and 6-min walking distance were significantly associated with CI (Zuccalà et al., 1997; Cacciatore et al., 1998; Pullicino and Hart, 2001; Baldasseroni et al., 2010; van Vliet et al., 2014). A possible explanation might be that other studies did not combine different cognitive domains into clusters, but analyzed performance in the respective domains directly. Therefore, clinical differences between patients with or without deficits in one domain might disappear after individual clustering.

The overrepresentation of women in the global deficit cluster is consistent with recent literature confirming the role of the female gender as a possible independent risk factor CI in HF patients (Volgman et al., 2019; Rossi et al., 2022). These differences are described as multifactorial, consisting of a stronger inflammatory reaction, higher reactivity of the autonomic system, and the amygdala as well as more frequent microvascular complaints or lack of cerebral perfusion (Volgman et al., 2019). Nevertheless, interpretations of gender differences must be made with caution, as the proportion of female patients of approximately 16% is strongly underrepresented in our cohort.

When considering cardiovascular risk factors (hypertension, diabetes mellitus, smoking, hyperlipidemia, and family history), the only differences were found in diabetes mellitus (higher proportion of women, *p* = 0.033). The genders did not differ in the number of interventions, the cause of heart failure (ischemic vs. non-ischemic), the frequency of stroke, or other comorbidities, such as chronic obstructive pulmonary disease, peripheral artery disease, and malignancies.

A gender-specific difference in HF patients is very present and absolutely plausible as published previously. The pathophysiological aspects and disease-specific entities appear to be fundamentally different. While women tend to develop microvascular dysfunction associated with HFpEF or conditions, such as Takotsubo or radiotherapy-associated cardiomyopathies, men are more likely to present with macrovascular conditions, which are more likely to manifest in myocardial infarction or coronary artery disease and may have a substantially different impact on cognition. Indeed, women with HF seem to suffer more severe impairment in cognition compared with men, which might have had a significant impact on cluster allocation with higher women share in the global deficits cluster (Lam et al., 2019; Volgman et al., 2019).

High frequencies of patients with valve operations in the “globally deficient” cluster may relate to described CI after aortic valve replacement (Zimpfer et al., 2002) and mitral valve replacement (Zhang et al., 2011). In these conditions, brain lesions were identified in the temporal lobe, a region also affected by chronic HF (Bokeriia et al., 2005; Frey et al., 2018). Our results are in contrast to some publications, which have found subcortical ischemia after heart valve surgery, but no reversible changes in cognition over a period of 4 months (Knipp et al., 2005), while we could not find correlates for valve surgery in brain morphological changes. In general, cognition is expected to change with aspects such as cardiopulmonary bypass time and possibly the etiology of valvular pathologies, which need further examination in patients with HF (Greaves et al., 2020). Further differentiation of valve defects certainly offers opportunities for more precise comparisons in future studies. However, in our cohort, the number of aortic valve stenosis (*p* = 0.705) did not differ while there was a higher proportion of mitral valve insufficiencies in the global deficient cluster (*p* = 0.015) when examined separately.

Hypertensive patients were highly represented in both our “intensity of attention” and the “globally deficient” cluster. CI is a well-known phenomenon associated with hypertension (Mercado and Hilsabeck, 2005; Reitz et al., 2007). Indeed, hypertension was also independently associated with the intensity of attention/executive function/psychomotor speed in another cohort of HF patients (Alosco et al., 2012a). Thus, controlled blood pressure might serve as a protective factor in our “non-deficient” cluster cohort. As an explanation, pathophysiological properties, such as neuroinflammation, disturbances of the blood–brain barrier, and chronical low perfusion, may be causative factors (Ungvari et al., 2021).

In fact, the selective influence of atrial fibrillation on cluster assignment and the prevalence of cognitive deficits within the clusters should be questioned, as the results are only very borderline significant and the clusters of no deficits and attention deficits are clinically very similar. In addition, our previous work has shown that although atrial fibrillation is a risk factor for cognitive impairment in general, it exists independently from HF-specific cognitive impairment (Frey et al., 2018).

### 4.4. Neurochemical and neuroradiological differences

Slightly higher serum levels of GFAP were seen in our “global deficient” cluster. Previous publications have already shown that GFAP is associated with CI (especially memory function) not only in Alzheimer's disease but also in chronic HF (Cicognola et al., 2021; Traub et al., 2022). In this line, neuronal biomarkers such as GFAP might be better suitable for predicting CI in HF patients compared with established HF measures. Moreover, we found slight differences in global brain volumes between cognitive clusters with the lowest volumes in the globally deficient cluster in a subset of patients, which is in line with our previously published finding in cognitively impaired HF patients (Frey et al., 2018). Furthermore, it was shown previously that lower brain volume influences both executive functions and memory (Fine et al., 2001; Duarte et al., 2006). It must be noted, that as total brain volume was not normalized to intracranial volume, reduced total brain volume in the “global deficits” group could reflect the fact that there were a higher proportion of females in this group.

Structural changes associated with cognitive decline in cardiac-compromised patients have been well-documented. One hypothesis is that differences in brain structure exist in principle compared with a normal cohort as found by us when comparing the whole HF cohort with a healthy cohort from the ASPS-Fam study from Graz, but not within the clusters when different clusters of HF patients are generated (Frey et al., 2018). Within our long-term observation, both cognition and morphological aspects of the brain remained stable over time, generating our hypothesis that there are certain risk factors that affect cerebral morphology and cognition at the onset or even before the clinical manifestation of heart failure but do not necessarily cause progression. Thus, we see the results of the cluster analysis as confirmatory and believe that we have been able to objectify certain risk factors for cognitive impairment. In the end, the amazing compensatory mechanisms of the brain are probably responsible for the fact that even patients with different cerebral morphology alterations can have equal cognitive performance.

### 4.5. Strengths and limitations

Our study comprised extensive neuropsychological workup, laboratory measurements, and MRI data, which are clear strengths of this investigation. The cluster method provides a more complete and holistic picture of the cognitively impaired patient compared with other studies that often only consider individual correlations of separate cognitive domains. Nevertheless, our cross-sectional evaluation remains limiting in regard to predicting the development of cognitive function over time. Due to the cross-sectional approach and the small differences found, the findings might be not clear enough to derive general recommendations but to define further research needs. Furthermore, the lack of a control group appears to be limiting, as the influence of confounding variables could not be comprehensively controlled. To some extent, the cognitively intact cluster can be seen as a reference group against the deficient ones in order to draw conclusions about the extent of certain differences. As the analysis used here was considered exploratory, no additional correction for multiple testing was performed. Thus, it must be taken into account that the inclusion of many variables may have led to false-positive results. Our results suggest that single HF parameters may not be sufficient to serve as a screening tool for CI in HF on their own. The solution could be in combining several markers covering more facets of HF, which needs further investigation.

## 5. Conclusion

Cluster analysis of patients with chronic HF revealed individual cognitive profiles with only marginal clinical, cardiological, and neuroradiological differences. It was interesting to see that selective deficits in the cognitive domain intensity of attention defined a separate cognitive cluster. Larger studies with a longitudinal analysis will be needed to identify the time-stability of cluster affiliation. Furthermore, the role of neuronal biomarkers and brain MRI awaits further evaluation. Associations between neuropsychological dimensions and their influencing factors may allow a better understanding of CI profiles and possible approaches to prevention in HF patients.

## Data availability statement

The raw data supporting the conclusions of this article will be made available by the authors, without undue reservation.

## Ethics statement

The studies involving human participants were reviewed and approved by Ethics Committee of the University Hospital Würzburg. The patients/participants provided their written informed consent to participate in this study.

## Author contributions

MP, SF, SS, GS, and AF: conceptualization. RS, GH, and MP: methodology. JT, DG, GH, MP, SS, and AF: formal analysis. RS, MV, GH, MP, and GS: resources. JT and DG: writing—original draft preparation. RS, GH, MP, SF, SS, GS, and AF: writing—review and editing. JT: visualization. SS, GS, and AF: supervision. All authors have read and agreed to the published version of the manuscript.
