# Supplementary material for: Profiles of cognitive impairment in chronic heart failure—A cluster analytic approach
Source: Front Hum Neurosci. 2023 Apr 20;17:1126553. doi: 10.3389/fnhum.2023.1126553 (PMC10157093; doi:10.3389/fnhum.2023.1126553)

frontiers

Supplementary Material

Profiles of cognitive impairment in chronic heart failure - a clusteranalytic approach

Content

Supplemental tables

Supplemental Table S1: Inclusion and exclusion criteria — page 2

Supplemental Table S2: Outcome of the neuropsychological test battery — page 3

Supplemental Table S3: Imaging protocol and sequence parameters — page 4

Supplemental Table S4: Extended clinical characteristics — page 5

Supplemental figures

Supplemental Figure S1: Spearman's correlation between cognitive domains — page 10

Supplemental Table S1: Inclusion and exclusion criteria for patients in the Cognition.Matters-HF study.

Inclusion criteria
1. Diagnosis of systolic heart failure (left ventricular ejection fraction ≤55%; any etiology or severity) or diastolic heart failure (any etiology or severity) present for at least 1 year up to a maximum of 15 years
2. Age ≥ 18 years
3. Written informed consent
**Exclusion criteria**
1. Previous stroke of any severity
2. Carotid artery stenosis >50%
3. Previous intracranial hemorrhage of any severity
4. Device implanted that impedes cranial magnet resonance imaging
5. Apparent psychiatric disease (especially depression or dementia)

Supplemental Table S2: Outcome variables of the neuropsychological test battery.

Test	Cognitive domain (specification)	Outcome variable (T-values)
**Test battery of Attentional Performance (TAP)**		
TAP Alertness	Intensity of attention or alertness (reaction times)	Median of reaction times with and without previous acoustic signal
TAP GoNoGo	Selectivity of attention (focusing, response inhibition)	Number of errors
TAP Divided attention	Selectivity of attention (dividing)	Number of omissions
TAP Incompatibility	Selectivity of attention (dividing, interference control)	Number of errors, incompatible condition (specific value visual field x hand)
**Visual and Verbal Memory Test (VVM2)**		
Map	Short- and medium-term visual memory	Number of correct crosses
Text	Short- and medium-term verbal memory	Number of correct responses
**Wechsler-Memory-Scale (WMS-R)**		
Digit span	Verbal working memory	Number of correctly remembered series
Block tapping span	Visual working memory	Number of correctly touched series
**Regensburger Word Fluency Test (RWT)**		
Lexically fluency	Verbal fluency (spontaneous cognitive flexibility)	Number of correct words
Lexically change of categories	Verbal fluency (spontaneous cognitive flexibility, shifting)	Number of correct words
**Hamasch-5-Point-Test (H5PT)**		
	Visual fluency (spontaneous cognitive flexibility)	Number of correct patterns

Supplemental Table S3: Imaging protocol, sequence parameters and specifications.

Sequence	repetition time [ms]	echo time [ms]	slice thickness [mm]	in-plane resolution [mm]	specifications
T1w FLASH*	260	4.67	3.0	0.7 x 0.6	2D GRE
T1w 3D TFL*	2530	2.15	1.0	1.0 x 1.0	MPRAGE, TI 1100 ms
T2w FLAIR*	9000	135	3.0	0.9 x 0.9	TI 2440 ms
T2w TSE*	6100	100	3.0	0.7 x 0.5	FA 120°
DWI	6500	91	3.0	1.9 x 1.9	b1 0 s/mm²; b2 1000 s/mm²
Localizers[1]	250	2.46	3.0	1.3 x 0.9	Triplanar
SV-Spectroscopy	2000	135	20[2]	(20 x 20)	L, R Parietal SVS
ASL Perfusion	2500	11	6	3.0 x 3.0	Pulsed; TI1 1800 ms; TI2 700 ms

ASL, arterial spin labeling; DWI, diffusion-weighted imaging; FA, flip angle; FLAIR, fluid-attenuated inversion recovery; FLASH, fast low-angle shot; GRE, gradient recalled echo; L, left; MPRAGE, magnetization-prepared rapid gradient-echo; R, right; SVS, single voxel spectroscopy; TE, echo time; TFL, turbo fast low-angle shot; TI, inversion time; TR, repetition time; TSE, turbo spin echo. [1] for Spectroscopy planning, [2] Single voxel dimensions.

Supplemental Table S4: Patient characteristics according to cognitive clusters.

Parameter	All patients (n=147)	No deficits (n=42)	Attention deficits (n=55)	Global deficits (n=50)	P-value
**Cognitive domains**					
Intensity of attention (T-score)	42 (37-47)	49 (46-53)	39 (35-42)	40 (36-44)	***<0.001
Memory (T-score)	45 (40-52)	46 (44-51)	52 (46-54)	39 (34-40)	***<0.001[W]
Executive functions (T-score)	45 (42-50)	47 (43-52)	47 (45-50)	41 (39-44)	***<0.001
**Morphometric measures**					
Age (years)	64 (56-72)	66 (54-75)	63 (54-70)	68 (58-74)	0.163
Female sex	23 (16)	4 (10)	5 (9)	14 (28)	*0.012
Body mass index (kg/m²)	28 (26-32)	28 (25-31)	29 (26-33)	28 (26-32)	0.603
Heart rate (bpm)	63 (58-70)	60 (57-71)	62 (57-67)	69 (59-76)	*0.035
Systolic blood pressure (mmHg)	136 (125-150)	135 (120-151)	138 (125-153)	137 (126-148)	0.775
Diastolic blood pressure (mmHg)	80 (75-89)	80 (75-85)	84 (73-90)	80 (75-88)	0.677
Mean arterial pressure (mmHg)	100 (92-109)	98 (90-107)	102 (93-110)	100 (93-106)	0.653
**Heart failure indicators**					
Years since diagnosis of heart failure	4 (1-10)	6 (1-11)	4 (1-9)	4 (1-10)	0.487
New York Heart Association class	2 (1-2)	2 (1-2)	2 (2-2)	2 (1-2)	0.624
6-minute walking test distance (m)	400 (340-460)	420 (360-480)	420 (350-460)	440 (305-480)	0.138
Peripheral edema	24 (16.4)	8 (19.5)	7 (12.7)	9 (18.0)	0.466
Fatigue / weakness	58 (39.7)	18 (43.9)	22 (21.8)	18 (36.0)	0.744
Stress dyspnoea	104 (71.2)	26 (63.4)	40 (72.7)	38 (76.0)	0.415
Ischemic heart failure	95 (64.6)	26 (61.9)	39 (70.9)	30 (60.0)	0.460
Regular physical activity	118 (80.3)	32 (76.2)	46 (83.6)	40 (80.0)	0.658
Guideline-based heart failure therapy	124 (84.4)	36 (85.7)	48 (87.3)	40 (80.0)	0.568
**Risk factors**					
Diabetes mellitus type II	43 (29.3)	11 (26.2)	19 (34.5)	13 (26.0)	0.551
Arterial hypertension	118 (80.3)	28 (66.7)	48 (87.3)	42 (84.0)	*0.029
Hyperlipidemia	106 (72.1)	33 (78.6)	43 (78.2)	30 (60.0)	0.063
Alcohol intake (drinks per week)	1.0 (0.0-5.0)	2.0 (0.0-3.8)	1.5 (0.0-7.0)	1.0 (0.0-2.0)	0.182
(Former) smoking	88 (59.9)	31 (73.8)	33 (60.0)	24 (48.0)	*0.042
**Pre-existent conditions**					
Coronary artery disease	99 (67.3)	29 (69.0)	39 (70.9)	31 (62.0)	0.600
History of myocardial infaction	79 (53.7)	24 (57.1)	34 (61.8)	21 (42.0)	0.110
Atrial fibrillation	34 (23.1)	14 (33.3)	7 (12.7)	13 (26.0)	*0.049
Primary valvular disease	9 (6.1)	3 (7.1)	2 (3.6)	4 (8.0)	0.614
Primary cardiomyopathy	24 (16.3)	6 (14.3)	7 (12.7)	11 (22.0)	0.401
Peripheral artery disease	14 (9.5)	3 (7.1)	5 (9.1)	6 (12.0)	0.725
Chronic obstructive pulmonary disease	20 (13.6)	4 (9.5)	9 (16.4)	7 (14.0)	0.619

Parameter	All patients (n=147)	No deficits (n=42)	Attention deficits (n=55)	Global deficits (n=50)	P-value
Malignoma	5 (3.4)	1 (2.4)	3 (5.5)	1 (2.0)	0.566
History of syncope	8 (5.4)	2 (4.8)	1 (1.8)	5 (10.0)	0.177
Sleep apnea	21 (14.3)	9 (21.4)	9 (16.4)	3 (6.0)	0.093
**Previous cardiovascular interventions**					
Coronary revascularization	70 (47.6)	21 (50.0)	30 (54.5)	19 (38.0)	0.288
Bypass operation	35 (23.8)	11 (26.2)	9 (16.4)	15 (30.0)	0.238
Valvular operation	10 (6.8)	0 (0.0)	2 (3.6)	8 (16.0)	**0.005
Reanimation / defibrillation	8 (5.4)	1 (2.4)	5 (9.1)	2 (4.0)	0.386
**Electrocardiogram**					
PQ time (ms)	179 (158-202)	177 (153-202)	186 (163-204)	175 (151-204)	0.724
QRS time (ms)	110 (99-128)	108 (95-127)	115 (102-131)	106 (98-122)	0.613
QT time (ms)	419 (400-448)	421 (395-446)	418 (398-449)	424 (401-448)	0.303
**Transthoracic echocardiography**					
LV end diastolic dimension (mm)	59.0 (56.0-64.0)	60.0 (57.0-67.0)	60.5 (56.3-64.0)	58.0 (54.0-62.0)	0.248
LV end systolic dimension (mm)	47.0 (42.0-51.0)	45.5 (42.0-49.8)	46.0 (40.5-51.0)	48.5 (41.8-52.8)	0.732
Left atrial endsystolic diameter (mm)	44.0 (39.3-47.0)	43.0 (39.3-46.8)	43.5 (40.0-46.0)	46.0 (39.0-48.8)	0.787
Septal wall end diastolic (mm)	11.0 (9.0-11.8)	10.0 (9.0-11.0)	11.0 (9.0-12.0)	11.0 (9.0-11.8)	0.298
Posterior wall end diastolic (mm)	10.0 (8.0-10.8)	9.0 (8.0-10.0)	10.0 (8.0-11.8)	10.0 (8.0-10.0)	0.794
Left atrial area (cm^2)	23.0 (20.3-28.0)	23.5 (20.3-26.5)	22.0 (19.3-27.8)	23.5 (22.0-29.8)	0.974
Left atrial volume (cm^3)	71.5 (61.3-99.3)	70.5 (60.0-88.0)	69.0 (62.3-99.3)	73.5 (61.3-111.3)	0.956
Left atrial volume index (ml/m^2)	35.7 (29.1-51.9)	35.5 (28.0-42.5)	34.2 (29.2-48.3)	41.3 (30.7-58.8)	0.808
Left ventricular ejection fraction (%)	44.0 (37.3-48.0)	43.5 (35.0-48.8)	45.0 (41.0-48.0)	39.0 (32.0-45.8)	0.222
LV volume end diastolic (ml)	126.5 (112.5-148.3)	121.5 (111.0-161.3)	125.5 (116.0-141.8)	131.0 (108.5-186.5)	0.474
LV volume end systolic (ml)	69.5 (60.3-90.0)	70.5 (53.5-92.0)	66.5 (62.3-77.3)	78.0 (59.5-125.5)	0.548
Right ventricular size (mm)	31.0 (28.0-35.0)	31.0 (28.0-33.8)	29.5 (27.3-33.8)	32.5 (27.3-36.8)	0.336
A wave (cm/s)	71.0 (60.3-98.3)	65.5 (61.0-81.5)	70.0 (60.5-94.5)	86.0 (60.0-122.0)	0.759
E wave (cm/s)	70.0 (52.5-90.0)	70.0 (54.3-86.0)	71.0 (60.0-93.8)	63.5 (37.0-91.5)	0.162
E/A ratio	0.9 (0.7-1.8)	0.8 (0.7-1.2)	0.9 (0.7-1.5)	1.4 (0.7-2.6)	0.292
Deceleration time (ms)	196.5 (148.3-236.0)	212.5 (154.8-242.0)	196.5 (151.8-229.0)	174.0 (142.8-235.0)	0.966

Parameter	All patients (n=147)	No deficits (n=42)	Attention deficits (n=55)	Global deficits (n=50)	P-value
Isovolumetric relaxation time (ms)	104.5 (90.5-118.0)	111.0 (103.3-115.5)	105.0 (93.0-117.5)	98.5 (83.0-127.3)	0.749
e‘ wave (cm/s)	7.0 (6.0-9.0)	6.5 (4.3-8.0)	7.5 (6.0-10.0)	7.3 (5.3-9.0)	0.654
a‘ wave (cm/s)	6.0 (4.0-8.0)	6.5 (5.0-8.0)	6.5 (4.3-9.5)	6.0 (3.0-9.0)	0.789
E/e‘ ratio	10.7 (7.8-13.6)	10.8 (8.2-13.9)	9.9 (7.0-12.6)	11.5 (8.8-14.9)	0.703
Trans-tricuspid gradient (mmHg)	26.5 (22.0-34.0)	24.0 (21.3-27.5)	26.5 (22.3-33.5)	31.5 (23.3-44.8)	*0.041
**Laboratory data**					
Alanine aminotransferase (U/l)	24.0 (18.0-32.0)	24.0 (19.5-33.0)	27.0 (19.0-34.5)	22.0 (16.8-29.5)	0.331
Albumine (g/dl)	4.60 (4.33-4.70)	4.60 (4.30-4.75)	4.60 (4.45-4.70)	4.50 (4.30-4.70)	0.279^{W}
Aspartate aminotransferase (U/l)	26.0 (22.0-31.0)	26.0 (21.8-29.5)	25.0 (21.0-30.5)	28.0 (22.7-33.3)	0.576
Cholesterol (mg/dl)	179 (152-209)	177 (154-222)	179 (154-198)	152 (183-218)	0.354
Creatinin kinase (U/l)	109 (77-154)	110 (75-150)	107 (87-156.5)	105 (64-173)	0.449
Creatinin kinase, muscle-brain (U/l)	16.4 (14.2-18.9)	17.3 (15.5-18.5)	16.3 (14.1-20.5)	16.3 (14.2-18.5)	0.793
C-reactive protein (mg/dl)	0.21 (0.09-0.51)	0.13 (0.09-0.40)	0.23 (0.10-0.47)	0.08 (0.29-0.73)	0.151^{W}
Estimated GFR (ml/min/1.73 m²)	67.5 (52.4-78.5)	66.0 (53.9-77.4)	69.0 (55.2-81.5)	63.7 (48.7-76.3)	0.179
Iron (µg/dl)	91 (68-114)	93.0 (73.5-120.5)	88.0 (67.0-107.0)	93.0 (62.5-119.0)	0.455
Ferritin (µg/l)	198 (95-325)	205 (114-365)	243 (122-342)	112 (64-220)	0.738
Fibrinogen (g/l)	3.40 (3.00-3.90)	3.40 (2.95-3.55)	3.40 (2.90-3.80)	3.60 (3.10-4.10)	0.207
γ-glutamyltransferase (U/l)	35.0 (24.0-62.0)	34.0 (24.0-49.1)	34.0 (23.5-64.7)	39.0 (25.5-69.5)	0.443
Hemoglobin (g/dl)	14.5 (13.6-15.4)	14.9 (14.1-15.8)	14.4 (14.0-15.0)	14.0 (13.1-15.1)	0.057^{W}
Uric acid (mg/dl)	6.60 (5.50-7.40)	6.60 (5.50-7.45)	6.60 (5.40-7.35)	6.60 (5.58-7.48)	0.906
Urea (mg/dl)	39.3 (32.9-48.8)	37.4 (30.3-49.2)	39.4 (33.1-49.4)	40.9 (34.5-48.6)	0.261
HbA1c (%)	6.00 (5.50-6.60)	5.90 (5.45-6.50)	6.00 (5.50-6.60)	6.00 (5.60-6.63)	0.133
High density lipoprotein (mg/dl)	46.0 (38.0-55.0)	50.0 (41.0-55.0)	45.0 (36.0-53.0)	45.0 (37.0-57.8)	0.682
Hematocrit (%)	42.5 (40.4-44.8)	43.8 (40.7-46.5)	42.0 (41.3-44.0)	41.5 (39.3-44.7)	0.175
Potassium (mmol/l)	4.50 (4.20-4.80)	4.50 (4.20-4.70)	4.50 (4.25-4.75)	4.65 (4.28-4.93)	0.143
Complement factor 3c (mg/dl)	120 (106-133)	119 (106-135)	122 (107-133)	118 (108-133)	0.937
Leukocytes (10³/µl)	6.90 (5.80-8.10)	7.25 (5.78-8.03)	7.3 (5.90-8.20)	6.6 (5.58-8.18)	0.864

Parameter	All patients (n=147)	No deficits (n=42)	Attention deficits (n=55)	Global deficits (n=50)	P-value
Mean corp. hemoglobin concentr. (g/dl)	33.9 (33.3-34.6)	34.0 (33.4-34.9)	34.0 (33.4-34.6)	33.7 (33.2-34.2)	*0.043
Mean corpuscular hemoglobin (pg)	30.6 (29.4-31.7)	30.6 (29.3-31.8)	30.7 (29.5-31.8)	30.4 (29.6-31.6)	0.798
Mean corpuscular volume (fl)	89.9 (87.3-93.3)	88.8 (87.0-92.9)	89.7 (87.0-93.7)	90.9 (87.6-93.7)	0.706
Natrium (mmol/l)	140 (138.0-141.0)	139 (138-141)	140 (138-141)	140 (138-141)	0.992
NT-proBNP (pg/ml)	612 (235-1736)	601 (254-1590)	431 (149-1080)	1033 (301-1889)	0.364
Triglycerides (mg/dl)	157 (112-220)	149 (88-202)	165 (121-225)	154 (103-239)	0.458
Thrombocytes (10^3/µl)	207 (173-260)	220 (164-264)	213 (175-268)	193 (165-248)	0.364
Transferrin (mg/dl)	267 (238-299)	267 (237-302)	260 (236-294)	274 (246-299)	0.933
Transferrin saturation (%)	23.8 (18.9-30.6)	24.8 (20.4-31-7)	22.6 (19.1-27.9)	24.4 (16.3-29.8)	0.624
Thyroid-stimulating hormone (mIU/l)	1.20 (0.80-1.95)	1.10 (0.75-1.75)	1.20 (0.80-2.00)	1.15 (0.90-2.18)	0.870
**Neuronal serum biomarkers**					
Glial fibrillary acidic protein (pg/ml)	247 (164-380)	226 (137-396)	212 (155-348)	307 (194-398)	0.048*
Neurofilament light chain (pg/ml)	26.2 (17.0-41.6)	22.5 (14.5-35.3)	27.5 (15.2-42.7)	28.5 (22.6-40.7)	0.127
Phosphorylated Tau Protein (pg/ml)	1.63 (1.08-2.50)	1.69 (1.04-2.36)	1.38 (0.98-2.40)	1.74 (1.22-3.01)	0.183
**Cerebral magnet resonance imaging**					
Lacunar infarction (%)	9 (6.7)	4 (9.7)	2 (3.8)	3 (6.3)	0.331
Territorial infarction (%)	2 (1.4)	2 (5.1)	0 (0.0)	0 (0.0)	0.081
Microinfarctions global (%)	(38)	17 (42)	22 (41)	16 (33)	0.810
Total brain volume (ml)	1184 (1116-1251)	1191 (1135-1264)	1198 (1137-1269)	1136 (1100-1225)	0.017*
Periventricular hyperintenity score (0-3)	1.00 (1.00-1.00)	1.00 (1.00)	1.00 (1.00)	1.00 (1.75)	0.818
Outer cerebral atrophy score (0-8)	3.00 (2.00-3.25)	3.00 (2.00-4.00)	3.00 (2.00-3.00)	3.00 (2.00-3.00)	0.778
Inner cerebral atrophy score (0-8)	3.00 (2.00-4.00)	3.00 (2.00-5.00)	3.00 (2.00-4.00)	3.00 (3.00-4.00)	0.835
Cerebral atrophy score total (0-8)	3.00 (2.50-4.00)	3.00 (2.00-4.75)	3.00 (2.25-4.00)	3.00 (2.50-3.50)	0.820
Hippocampal atrophy right (0-4)	2.00 (1.00-3.00)	2.00 (1.00-2.00)	2.00 (1.00-2.00)	2.00 (1.00-3.00)	0.233
Hippocampal atrophy left (0-4)	2.00 (1.00-3.00)	2.00 (1.00-3.00)	2.00 (1.00-3.00)	2.50 (2.00-3.00)	0.221
Hippocampal atrophy total (0-4)	2.00 (1.38-2.63)	2.00 (1.00-2.50)	2.00 (1.00-2.50)	2.00 (1.50-3.00)	0.179
Pathological Schelten's score	106 (74.6)	28 (68.3)	37 (69.8)	41 (85.4)	0.107
**Medication**					

Parameter	All patients (n=147)	No deficits (n=42)	Attention deficits (n=55)	Global deficits (n=50)	P-value
Angiotensin-converting enzyme inhibitors	86 (59)	24 (57)	33 (60)	29 (58)	0.957
Beta-blockers	131 (89)	38 (91)	48 (87)	45 (90)	0.855
Aldosterone antagonists	55 (37)	16 (38)	20 (36)	19 (38)	0.979
Cardiac glycosids	21 (14)	4 (10)	7 (13)	10 (20)	0.330
Thiazids	23 (16)	4 (10)	10 (18)	9 (18)	0.434
Loop Diuretics	81 (55)	23 (55)	31 (56)	27 (54)	0.970
Potassium-sparing diuretics	(0)	0 (0)	0 (0)	1 (2)	0.377
Nitrates	3 (2)	0 (0)	2 (4)	1 (2)	0.455
Calcium antagonists)	16 (11)	4 (10)	8 (15)	4 (8)	0.530
Antiarrhythmics	9 (6)	3 (7)	5 (9)	1 (2)	0.302
Statins	101 (69)	29 (69)	41 (75)	31 (62)	0.383
Acetylsalicylic acid	79 (54)	19 (45)	35 (63.6)	25 (50.0)	0.160
Other platelet inhibitor	21 (14.3)	9 (21.4)	8 (14.5)	4 (8.0)	0.186
Vitamin K antagonists	46 (31.3)	16 (38.1)	11 (20.0)	19 (38.0)	0.074
Insulin	14 (9.5)	3 (7.1)	8 (14.5)	3 (6.0)	0.272
Oral antidiabetics	35 (23.8)	8 (19.0)	16 (29.1)	11 (22.0)	0.482
Pulmonal inhalatives	14 (9.5)	3 (7.1)	8 (14.5)	3 (6.)	0.272
Antidepressants	11 (7.5)	5 (11.9)	3 (5.5)	3 (6.0)	0.433
Sleeping medication	4 (2.7)	2 (4.8)	1 (1.8)	1 (2.0)	0.629
Allopurinol	33 (22.4)	9 (21.4)	12 (21.8)	12 (24.0)	0.948
Analgesics	4 (2.7)	0 (0.0)	0 (0.0)	4 (8.0)	*0.019

Values are given as median (interquartile range) or in absolute numbers (percent); ACE, angiotensin-converting enzyme; LV, left ventricle; ^{W} = Welch test was applied due to missing homogeneity of variances

<u>**Supplemental figure 1:**</u> **Spearman's correlation between cognitive domains.** ρ = Spearman's rho

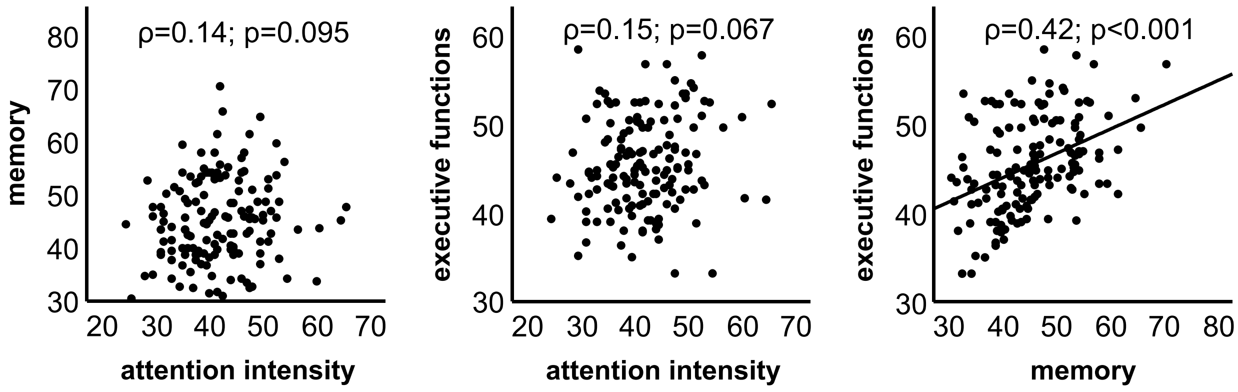

Supplement: Supplementary file 1 [file Data_Sheet_1.pdf]
